# Investigation on Hamburg Wheel-Tracking Device Stripping Performance Properties of Recycled Hot-Mix Asphalt Mixtures

**DOI:** 10.3390/ma13214704

**Published:** 2020-10-22

**Authors:** Waqas Rafiq, Madzlan Bin Napiah, Muslich Hartadi Sutanto, Wesam Salah Alaloul, Zarisha Nadia Binti Zabri, Muhammad Imran Khan, Muhammad Ali Musarat

**Affiliations:** 1 Department of Civil and Environment Engineering, Universiti Teknologi PETRONAS, Perak 32610, Malaysia; madzlan_napiah@utp.edu.my (M.B.N.); muslich.sutanto@utp.edu.my (M.H.S.); wesam.alaloul@utp.edu.my (W.S.A.); zarishazabri@gmail.com (Z.N.B.Z.); muhammad_17007177@utp.edu.my (M.I.K.); muhammad_19000316@utp.edu.my (M.A.M.); 2 Department of Civil Engineering, COMSATS University Islamabad, Wah Cantt 47040, Pakistan

**Keywords:** recycled asphalt pavement, Hamburg Wheel Tracking Device, rutting, stripping performance

## Abstract

Moisture damage in hot mix asphalt pavements is a periodic but persistent problem nowadays, even though laboratory testing is performed to identify different moisture-susceptible mixtures. In this study, a Hamburg Wheel Tracking device (HWTD) was used for rutting tests which were conducted on control and a high percentage of recycled asphalt pavement (RAP), i.e., 30%, 50% and 100% of virgin mixtures, under air dry and water-immersed conditions. Similarly, the extracted bitumen from RAP was tested for binder physical properties. Results showed that the asphalt mixtures containing RAP have less rut depth as compared to the control mix both in air dry and immersion conditions and hence showed better anti-rutting properties and moisture stability. Stripping performance of control and RAP containing mixtures was also checked, concluding that the RAP mixture was greatly dependent on the interaction between the binder (virgin plus aged) and aggregates.

## 1. Introduction

In developing countries, transportation infrastructure highly impacts prosperity. Most of the public and cargo movements are passed through roads, which is the major resource of transportation. When roads are designed as per standards and specifications, it is more durable and have an anticipated serve life. The role of recycled asphalt pavement (RAP) is remarkable and being enhanced with time, as it not only fulfils the requirements but is also cost and environment efficient. There are various methods for asphalt recycling where binder and aggregate are main materials which are reused to build pavement [1,2,3].

With the passage of time, asphalt pavement tends to deteriorate due to traffic loading and environmental stresses which causes the breakdown of bond between aggregates and binder. The deterioration could be due to improper compaction efforts of different layers, mixing and compaction temperature and poor drainage, etc. Researchers are looking for RAP incorporated mixtures due to their potential benefits against distresses [4].

Moreover, researchers in the asphalt industry are looking for innovative methods and technologies to ensure sustainability, less greenhouse gases and efficiency. Construction sectors from recent few years are reutilizing the materials based on new technologies that are being adopted. The consumption of RAP in hot asphalt mixtures can release the pressure on the utilization of natural aggregate resources and result in less harm to nature. RAP in the construction sector is being utilized in other areas as well. RAP is being used in embankment material and only in the form of aggregate in recycling technologies [5,6]. With the addition of RAP to bituminous mixtures, there is a considerable reduction in pavement material by 23%, reducing the burden on the environment [7]. Besides this, RAP also helps in material cost saving. Finding and procuring fresh and quality aggregates are becoming difficult in many regions. Thus, the incorporation of RAP can reduce the cost and allows the authorities to rehabilitate more roadways within the limited budget. Highway agencies allow the incorporation of RAP in wearing course, but not above 30% [6,7,8,9,10].

High RAP content material should be well characterized for both mix design and quality control parameters. Multiple locations around the RAP stockpile should be selected for sample collection by using the back dragging technique for the determination of binder properties and variability in milled material gradation [11,12]. Transportation department of US has increased the usage of RAP into pavements due to economic and environmental concerns. The higher the amount, greater is the impact on structural performance and durability due to RAP usage [13]. When RAP amount is high in HMA, most of the distresses are associated with aged binder which impacts performance properties such as: less elastic binder in RAP mixtures, increases mixture stiffness and can cause fatigue damage and low temperature brittleness, permanent deformation and fracture characteristics. Highway and Government agencies are not allowing very high RAP content due to aforementioned reasons highlighted [14].

Various studies have been conducted to overcome those arises issues link to the performance of materials utilization in road construction. Depleting and declining quality of resources had triggered the industry in seeking alternatives for these issues. Currently, the competition and dwindling maintenance are at peak, particularly in road rehabilitation [15]. However, according to the Federal Highway Administration, US Department of Transportation reported an average 33% of RAP usage in HMA. Rejuvenators and antistripping agents are being used in addition with RAP mixtures as modifiers to provide improved performance and rheological properties [12,16]. A study was conducted on Superpave with 19 mm nominal maximum aggregate size along with different percentage of RAP material (15%, 25% and 40%), where an increase of 25% and 40% of voids in mineral aggregate (VMA) and voids filled with asphalt (VFA) was seen. It was revealed that pre-heating on the volumetric properties also influenced the performance properties. These trends likely caused by the combined effect of gradation, volumetric properties and asphalt content [17,18].

In another study, 20%, 30% and 40% recycled mixtures and fractioned RAP were used to find how high RAP percentage influences volumetric properties of the mixtures. Significantly various values of volumetric properties were found which were further utilized [19,20,21]. Research showed that using high RAP content up to 50% bituminous mixtures become brittle, and result in premature crack failure. Using rejuvenators to tackle the cracking of asphalt mixtures with a high percentage of RAP content was considered. With rejuvenator volumetric properties of asphalt mixture air void, VMA and dust to binder (d/b) ratio were determined. The result showed that volumetric properties changed significantly when a rejuvenator in line with different mixing procedures was used [22,23,24]. In the past few years, traffic volume has increased dramatically, causing premature pavement distress which is the major issue in most of the countries. Rutting in flexible pavement occurs due to many factors such as: binder properties, air void, environmental conditions, compaction level, asphalt mixture stability, etc. [25,26,27]. Studies have been carried out for the evaluation of permanent deformations like rutting and moisture damage when repeated loading by a mechanized wheel was subjected to asphalt specimens. By corelating the rutting susceptibility and mixture properties, it is possible to get a good idea of the optimized mixture design to be chosen by road design experts [28,29,30,31]. For investigating the rut depth effect, aggregate size regression models were framed for determining the independent variable importance with output of rut value of asphalt specimens which comes under cycling loading [32,33,34,35,36].

In hot mix asphalt loss of the integrity occurs that weakens the adhesive bonding between aggregate and bitumen, known as stripping, which generally begins in the bottom of the HMA layer and then moves upward. This phenomena cause distresses like rutting, shoving, corrugations, raveling and cracking that developed in wheel path due to gradual loss of strength over a period [37,38]. Many other causes of stripping are possible, inadequate drainage or subsurface drainage is the primary contributor for that. Capillary action due to water table, surface runoff and seepage from surrounding vicinity induce the moisture that can infiltrate into the HMA layers. That may cause internal pour pressure and weakens the aggregate and asphalt bond if careful consideration to air voids are not given [39,40]. Moisture damage is one of the major problems in Malaysian asphalt pavements because of the number of mechanisms, such as cohesion failure induced by moisture within the binder, within the aggregates, and adhesion failure between aggregates and binder. Regarding moisture sensitivity of hot mix asphalt, many traditional asphalt concrete testing is done underwater, which includes Hamburg wheel-tracking testing and asphalt pavement analyzer testing. Investigations regarding rutting performance associated with moisture damage have also been adopted by various researchers. Moisture sensitivity of asphalt concrete samples has also been evaluated using the standard method for testing the resistance of compacted HMA to moisture-induced damage. Many researchers employed this technique for assessing moisture sensitivity of various mixtures and materials, but its correlation with actual performance in the field and laboratory evaluation is very low. That is why HWTD testing has been utilized in this research, as it is relatively simple to conduct and also provides the rutting potential of mixes by measuring sample rut depth.

Therefore, this research study addresses the volumetric issues induced by the inclusion of high percentages of RAP in HMA. The effect of aged and virgin binder blends on binder properties and performance of HMAs with high RAP content were examined. The results were presented herein, followed by evaluating the stripping resilience of high percentages of Recycled Asphalt Pavement mix (up to 100%), using Hamburg Wheel Tracking Device at air dry and under immersed mode at a high temperature. The influence of aggregate strength and durability on the performance of the bituminous mix in the HWTD test parameters, which included rutting, the slope of the rutting curve and different areas beneath the rutting curve at specific cycles were also discussed.

## 2. Materials and Methods

### 2.1. Materials

**Figure 1:**
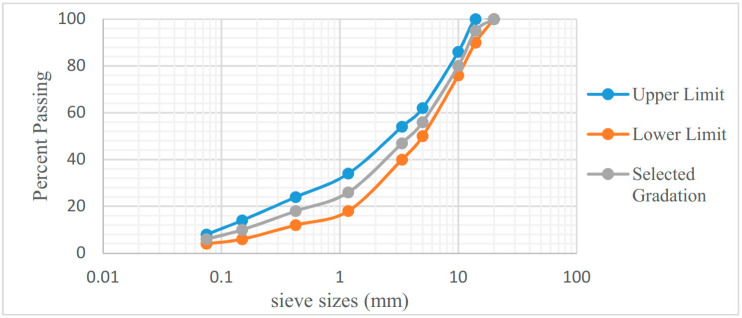
Gradation plot with JKR-specified limits.

According to Jabatan Kerja Raya (JKR) specification for flexible road works Malaysia, asphalt concrete (AC-14) wearing course gradation was selected for preparing dense graded surface course mixes [41]. Crushed granite aggregate was used as coarse aggregate and procured from Sunway quarry located in Ipoh, Malaysia. River sand was used as fine aggregate, obtained from Tronoh. Bitumen of 60/70 penetration grade was obtained from PETRONAS refinery. RAP in form of milled material was brought from Kamunting Premix Plant, Malaysia which was brought there from PLUS highway. The selected aggregate gradation for determining the optimum asphalt binder is plotted in Figure 1 along with the specified limits of AC-14 as suggested by JKR.

### 2.2. Binder Extraction and Determination of Binder Content

**Figure 2:**
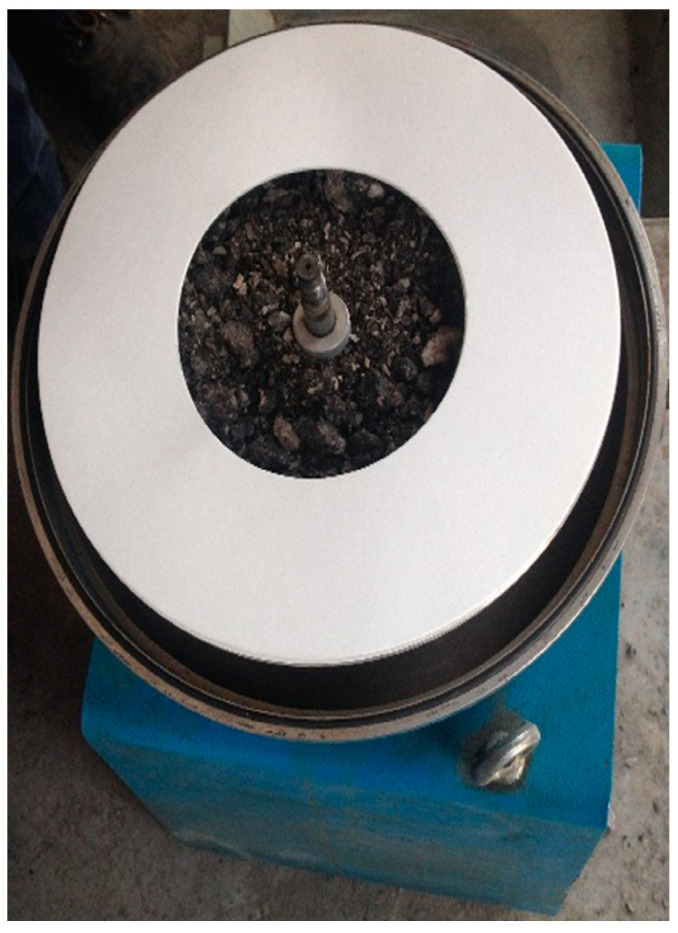
RAP material in Centrifuge Extractor.

AASTHO T 319 and ASTM D7906 procedures of asphalt extraction were used to determined binder content present in RAP material [42]. As per AASHTO standards, the methylene chloride solvent was used for extraction of bitumen from RAP material as shown in Figure 2.

**Figure 3:**
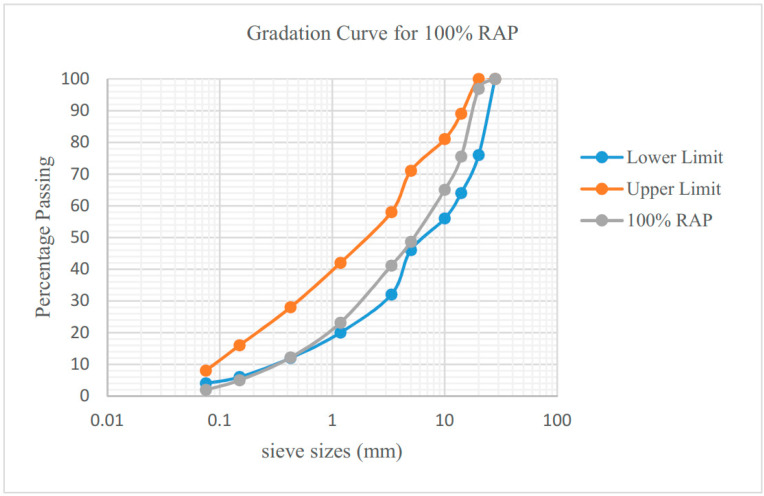
Extracted RAP gradation Curve.

The extracted binder content from RAP was found to be 4.73% by weight of the mixture after aging on site. The particle size distribution of RAP aggregate was evaluated through a sieve analysis test in accordance to AASHTO T27 [43] and the results are shown in Figure 3.

The RAP samples extracted shows the aggregate retained at sieve size of 20 mm. Hence, the RAP gradation curve conforming to the proposed blended mix for Type 2 of JKR was prepared. This gradation is set as the benchmark for the specification complied when RAP is added with the HMA mix. According to JKR, the gradation limits for a blended mix for Type 2 are shown in Table 1.

### 2.3. Physical Properties of the Virgin and Extracted Binders

The standard engineering tests have been performed, where bitumen penetration was assessed in compliance with ASTM D5 [44]. The ductility of asphalt material was determined by ASTM D113, where Ring & Ball softening point was assessed in accordance with ASTM D36 [45]. Rotational Viscometer was used to obtain the viscosity of binder as per ASTM D4402 [46]. A penetration grade of virgin bitumen 60/70 at 25 °C and softening point of 49 °C temperature was used as a control reference [47,48,49]. Penetration index (PI) shows the temperature vulnerability of bitumen. Higher -PI bitumen shows the softness and is used in regions with low temperature. Lower -PI bitumen displays hardness and brittleness, low flowability and high rate of cracking susceptibility so are employed in regions with high temperature [50,51,52]. The properties of the blended mix depend on the properties of the binder (RAP binder plus Virgin). The stiffness of the binder increases with increasing RAP percentage [53]. To cater for stiffness issue many studies proposed that soft HMA can be produced by combining the RAP with a soft penetration grade binder [54,55].

### 2.4. Marshall Mixture Preparation

The Marshall mix designs of virgin mix with 60/70 bitumen penetration in HMA samples were conducted. The volumetric properties included bulk unit weight (BUW), voids in mix (VIM), voids in mineral aggregate (VMA), voids filled with bitumen (VFB) were then enumerated for the determination of optimum binder content (OBC) for the control mix. Marshall mix design of the virgin mix for HMA with 0.5% bitumen content increment was carried out corresponding stability (S), flow (F) and volumetric properties are listed in Table 2.

### 2.5. Mixture Preparation Containing RAP

The RAP samples were prepared alongside with the gradation and binder properties. For this study, the test was initiated with 0%, 30%, 50% and 100% RAP in hot mix asphalt (HMA). Progressing of RAP aggregate involves many steps to make better performing material that can be used in high percentages and meeting the required high quality standards of asphalt mixtures. Sieves of different sizes are used for this process to get the aggregate of different sizes [56,57]. The gradation curve for the blended mix was prepared following the mix design proposed by JKR Malaysia. The percentage of binder content was adjusted accordingly after obtaining the OBC of the control mix. The RAP samples extracted shows the aggregate retained at the sieve size of 20 mm. Hence, the RAP gradation curve conforming to the proposed blended mix for Type 2 was prepared. This gradation is set as the benchmark for the specification complied when RAP is added with the HMA mix. The composition of the projected mix sample of 1200 g containing variations of RAP percentage was prepared, as shown in Table 3.

**Figure 4:**
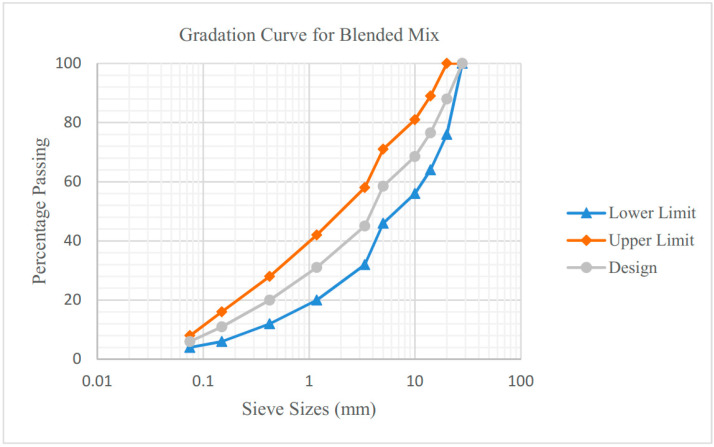
Gradation curve of blended mix.

Figure 4 shows the gradation curve prepared of the blended mix for RAP variations mix. The middle line shows the design limit which falls in between the upper and lower limit. This curve was set as the standard specification, as mentioned by JKR/SPJ/2008-S4. However, the gradation and properties from RAP may be subjected to change due to the aggregates breakdown or loss in the oven during heating [58].

**Figure 5:**
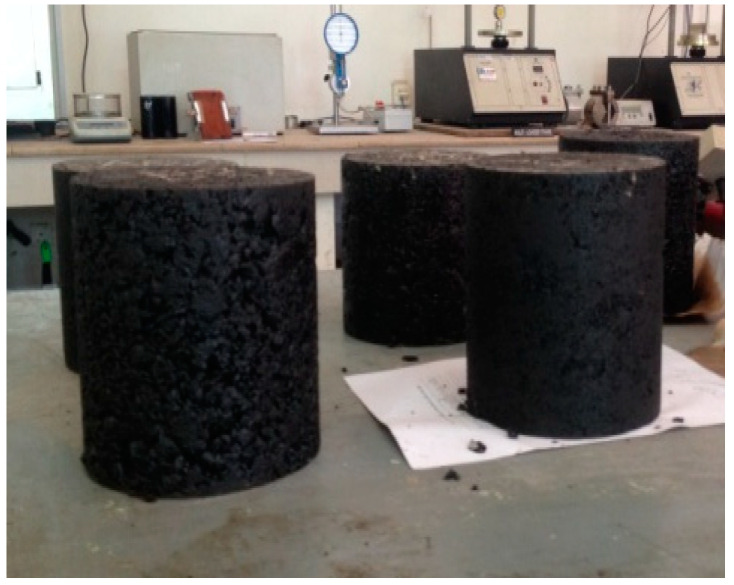
Specimens prepared with Gyratory Compactor.

**Figure 6:**
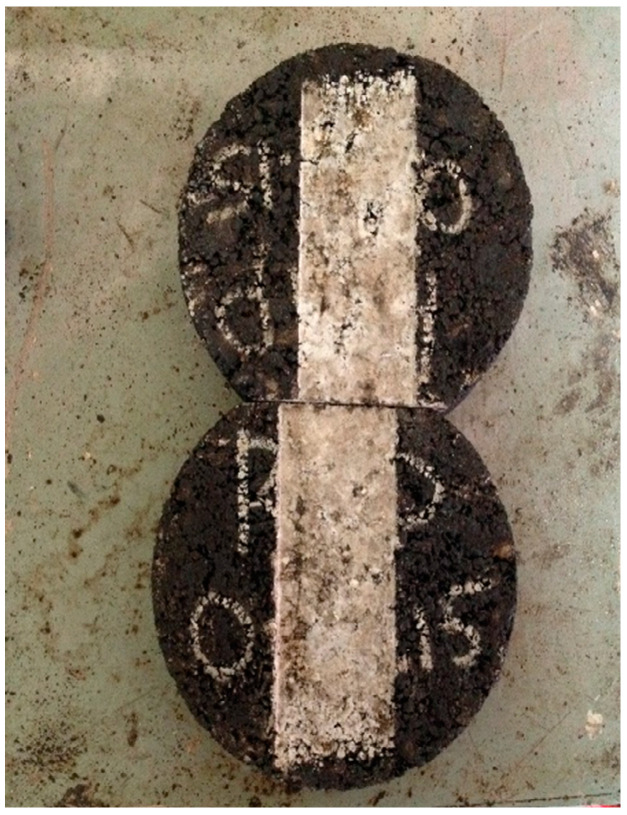
Saw-cut specimens for HWTD.

Compaction of specimen was done by using 125 number of gyrations of gyratory compactor. While preparing gyratory specimens selected RAP percentages were used. For both dry and wet conditions of Hamburg wheel tracker test three samples were prepared for each of the RAP containing samples. Extracted samples from the gyratory mold are shown in Figure 5. For HWTD mold, gyratory samples were saw cut to a standard height of 1.5 inches from both ends, as shown in Figure 6.

### 2.6. Hamburg Wheel Tracking Test

The HWTD test can be utilized in two modes, dry condition and submerged condition. Standard wheel load accompanied by various temperature conditions is used to evaluate the rutting resistance and moisture effect of mixtures [59,60,61]. The HWTD wheel load consists of 158 lb (705 N), 0.73 MPa of contact stress and 38 inch of contact area. The contact area of the wheel increases with increasing number of wheel passes. The wheel tracking test was performed on the air dry and wet conditioned submerged in water specimens at 50 °C for 20,000 passes of 158 lb of steel wheel or until 12.5 mm of deformation. Hamburg Wheel Tracking Device test outputs include different points which are defined as post-compaction consolidation, creep slope, stripping slope and stripping inflection point [62]. Stripping inflection point and stripping slope are linked with moisture resistance of hot mix asphalt. Stripping inflection point is the intersection point of the creep slope and stripping slope, in actual is the number of passes at that point. Stripping slope is after stripping inflection point and is an inverse rate of deformation and continues till the end of the test [34].

## 3. Results and Discussion

### 3.1. Penetration, Softening Point, Ductility and Penetration Index

The penetration, softening point, ductility, penetration index and viscosity results of the control and RAP modified asphalt binder samples are shown in Table 4.

**Figure 7:**
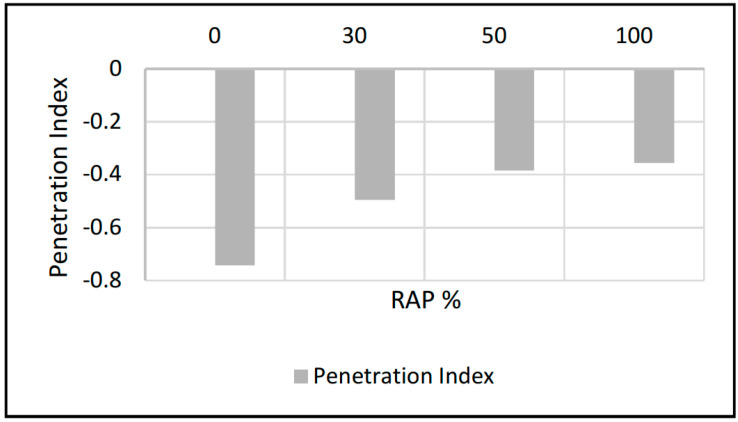
Penetration Index versus RAP content.

As expected, penetration value decreased as the RAP percentage increased, values of penetration decreased considerably to 66% from control to 100% RAP containing sample. Softening point temperature increased from control to 100% RAP containing sample up to 25%. Ductility reduced to 85% from control to 100% RAP containing sample. Viscosity values increased from control to 100% RAP containing samples up to 64% at 135 °C and 71% at 160 °C. The viscosity values showed that the workability of 100% RAP containing mixtures decreased, whereas penetration index (PI) showed the temperature vulnerability of bitumen. Figure 7 showed that the higher -PI value is because of the virgin or softer binder which was 60/70 grade. When RAP binder percentages increased, lower -PI bitumen was found showing hardness, brittleness and low flowability. It was observed that the properties of the blended mix depend on the properties of the binder (RAP binder plus virgin).

### 3.2. Marshall Properties of Control Mix and RAP Samples

Marshall test was utilized to check the effect of RAP percentages on the stability and volumetric properties of asphalt mixtures. The test was performed at optimum asphalt content of 5%. The RAP gradation blended mix as discussed earlier was used to prepare HMA samples. Volumetric results showed that air voids, VMA and VFA were showing variability due to the addition of higher RAP content. The standard deviation (std) was also calculated for each volumetric property for the control mix and RAP containing samples. The values showed that there is not much variation among the samples, which indicates the consistency of the obtained results. Stability of samples from control to 100% RAP containing samples was greater than JKR specification of 8 kN. With the increase in RAP material stability of 100% RAP sample increased to 45% as compared to the control sample showing a lot of stiffness and hardness. The flow of samples was also increased because of the hardness in the sample but was in the range of JKR specifications. The results of the volumetric properties such as BUW, VIM, VMA, VFB, stability and flow are tabulated in Table 5.

### 3.3. Wheel Tracker Rutting Damage Results under Air Dry Mode

**Figure 8:**
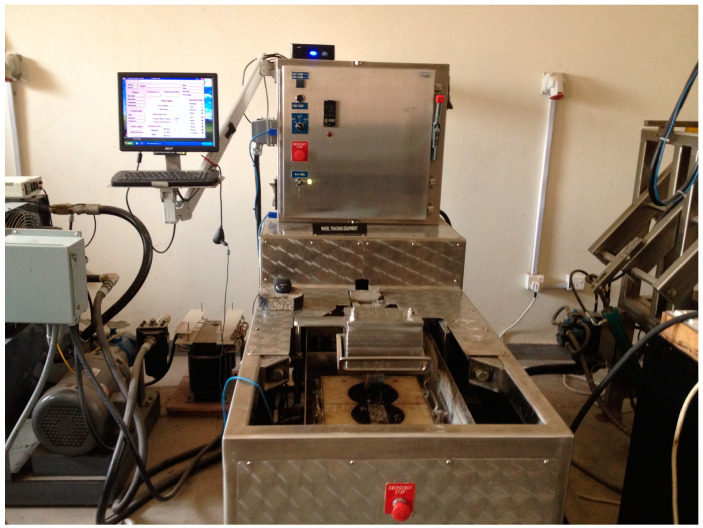
Hamburg Wheel Tracking Device and specimen setup.

Controlled and RAP mixtures were evaluated for rutting at air dry condition specimens with 4% air voids was selected at the test temperature of 30 °C. For conditioning and maintaining temperature, all specimens were equally placed in the HWTD at the test temperature for 8 h before testing. The test setup and arrangement of specimens are shown in Figure 8.

**Figure 9:**
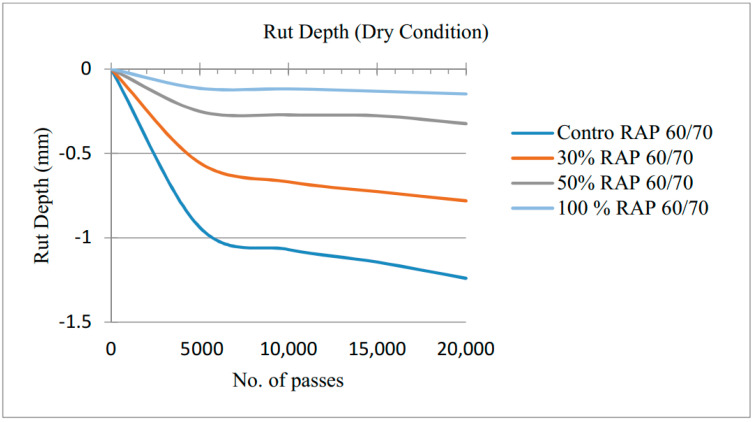
Rut depths under air-dry conditions.

The results of rut depth are demonstrated in Figure 9. The rut depth of −1.24 mm for control mix, −0.79 mm for 30% RAP, −0.32 mm for 50% RAP and −0.15 mm for 100% RAP obtained after 20,000 wheel passes. The addition of RAP having aged recycled asphalt binder showing the lower penetration binder grade, which increased the viscosity as depicted in virgin RAP binder blends, resulted in decreasing the rutting. Stiffness, which is a major concern in RAP mixtures, is due to frequent oxidation which changes the constituents of RAP binder and fades away the viscoelastic properties. Moreover, the consequences of changes in constituents, i.e., the asphaltenes to maltenes ratio in aged asphalt binders, cause the RAP stiffer in nature. As the amount of RAP increases the decrease in rutting was observed 36% with 30% RAP, 74% with 50% and 88% with 100% RAP showing the greater stiffness in HMA samples. The rut depth below 12.5 mm was considered as an acceptable range, control as well as RAP containing samples were below this value.

### 3.4. Wheel Tracker Moisture Damage Results under Wet Mode

**Figure 10:**
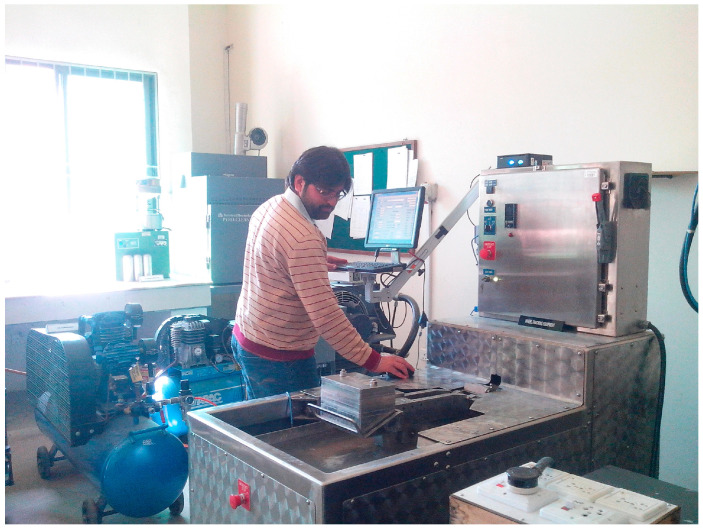
Specimen in water during submerged mode.

Under immersion conditions, the controlled and RAP containing specimens with the air voids of 4% were tested, respectively; the test temperature was 50 °C; the arrangement of specimen submerged in water during the test is shown in Figure 10.

**Figure 11:**
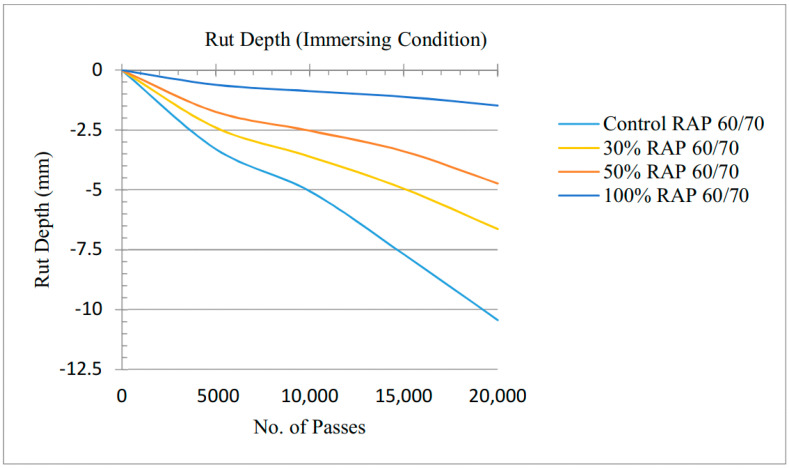
Rutting depths under immersing conditions.

For conditioning and maintaining temperature, equally all specimens were placed in the HWTD at the test temperature for 8 h before testing. Figure 11 demonstrates that rut depth is −10.5 mm for control specimen, −6 mm for 30% RAP, −4.8 mm for 50% RAP and −1.5 mm for 100%. The same pattern of rut decrease was found as RAP percentage increased 43% from 30% RAP, 54% from 50% RAP and 86% from 100% RAP samples. The results showed that the mixtures containing RAP from 10% to 100% have a rut depth of well below 12.5 mm and lies within the acceptable range. This is due to more stiffness of RAP mixtures as compared to virgin mixtures.

**Figure 12:**
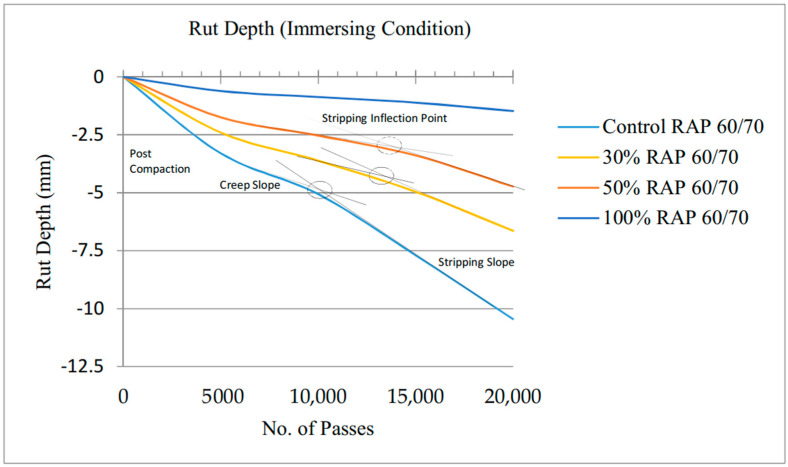
HWTD test result for Control and RAP-containing mixes.

Stripping inflection point for control mix is 10,000 passes, 13,200 for 30% RAP content, 13,800 for 50% RAP content and no stripping inflection point for 100% RAP as shown in Figure 12. The report compiled by the Colorado Department of Transportation (CDOT) reported that stripping inflection point and the stripping slope did clearly distinguish between the stripping performances. The stripping inflection point correlated with the various levels of expected pavement performance. As a rule of thumb, a stripping point greater than 14,000 passes may indicate good pavement performance that has an expected life of 10 to 15 years. A stripping point above 10,000 passes indicates the routine maintenance before the design life is reached [62].

To achieve hot mix asphalt mixtures containing a higher percentage of RAP, comprehensive binder’s blend design should be performed which provide the vital inputs for the mix design. While using JKR specification of flexible pavement for Malaysia, performance parameters are showing stiffness in the asphalt mixtures as depicted in the results, conventional mix design methodology for designing 100% RAP mixtures must be improved, particularly in respect to binder content calculation. The stripping performance results of mixtures provides basic to modify binder blend’s formulas for the production of asphalt mixtures incorporating 100% RAP. With this attention, the methodology followed in the current research is being used by asphalt technologists to maximize RAP usage having better control in the mixture quality. RAP should be handled carefully in the required fractions, as done in this research, to control the excess fine material during mixture gradation selection. Moreover, the environmental conditions affect the Malaysian roads, causing severe moisture damage because of average precipitation of the 250 mm per month. This study is also helpful for designing mixtures in the countries which have the same tropical weather as Malaysia.

## 4. Conclusions

The stripping performance of HMA pavements is greatly dependent on the bond between the binder and aggregates and the quality of the aggregate components in the HMA, which is a real-time problem in Malaysian pavements due to tropical weather. The HWTD with air dry and wet modes was used to evaluate the stripping performance of HMA mixtures containing RAP. If an HMA is showing less performance, testing the asphalt cement (virgin and aged) and aggregate (fresh and aged) portions of the mix can designate areas for potential upgrading of the mix. Therefore, based on the results and foregone discussions, the following conclusions are drawn:

- The addition of aged binder (extracted from RAP) causes a decline in the Penetration Index, indicating more brittleness and more susceptible to cracking. The Penetration Index of the virgin and binder containing 30%, 50% and 100% aged binder keeps on decreasing from 0.74 to 0.35.
- Viscosity of binder increased with addition of RAP percentages from 0.4 to 1.1 Pa∙s at 135 °C and from 0.10 to 0.34 Pa∙s at 160 °C, showing stiffness due to aging in the binder.
- Volumetric results showed that with mixtures with RAP content of more than 30%, careful consideration should be given to the asphalt binder grade to be added with recycled asphalt mixture in line with state specification designed by transportation authorities.
- Asphalt mixtures containing RAP performed well based on its Marshall stability properties. RAP with 100% utilization has showcased the stability of 22.89 kN as compared to the control mix stability of 12.69 kN.
- The Stripping performance is judged by stripping inflection point of the mixtures; the lower the stripping inflection point, the worse the stripping will be in the field. The stripping inflection point is at 10,000 passes for control mixtures, 13,200 for 30% RAP, 13,800 for 50% RAP and no stripping point is observed for 100% RAP.

## Figures and Tables

**Table 1:** Aggregate for blended mix by JKR.

Mix Designation	Type 2
BS Sieve Size (mm)	Percentage Passing by Weight
28	100
20	76–100
14	64–89
10	56–81
5	46–71
3.350	32–58
1.180	20–42
0.425	12–28
0.150	6–16
0.075	4–8

**Table 2:** Marshall properties of the virgin mix for HMA.

Bitumen Content	BUW	VIM (%)	VMA (%)	VFB (%)	S (kN)	F (mm)
4.0	2.408	4.09	14.82	63.10	16.49	2.74
4.5	2.418	3.90	14.92	70.80	17.10	3.23
5.0	2.424	3.39	15.16	77.63	14.83	3.43
5.5	2.427	3.20	15.49	83.68	12.10	3.31
6.0	2.424	3.03	16.03	88.12	9.45	3.11

**Table 3:** Composition of RAP variation containing in HMA mix.

Sieve Size (mm)	Average RAP	% Retained	% Passing	RAP/HMA Mass Retained (g)							
				100/0		50/50		30/70		0/100	
				100	0	50	50	30	70	0	100
28	-	100	0	-	-	-	-	-	-	-	-
20	14.93	3.13	96.87	37.61	0.00	18.80	18.80	11.28	26.33	0.00	37.61
14	101.97	21.40	75.47	256.79	0.00	128.39	128.39	77.04	179.75	0.00	256.79
10	49.70	10.43	65.04	125.16	0.00	62.58	62.58	37.55	87.61	0.00	125.16
5	78.17	16.40	48.63	196.85	0.00	98.43	98.43	59.06	137.80	0.00	196.85
3.350	35.67	7.49	41.15	89.82	0.00	44.91	44.91	26.95	62.88	0.00	89.82
1.180	85.80	18.01	23.14	216.08	0.00	108.04	108.04	64.82	151.25	0.00	216.08
0.425	52.40	11.00	12.14	131.96	0.00	65.98	65.98	39.59	92.37	0.00	131.96
0.150	34.03	7.14	5.00	85.71	0.00	42.85	42.85	25.71	60.00	0.00	85.71
0.075	14.50	3.04	1.96	36.52	0.00	18.26	18.26	10.95	25.56	0.00	36.52
Pan	9.33	1.96	0.00	23.50	0.00	11.75	11.75	7.05	16.45	0.00	23.50

**Table 4:** Penetration, Softening Point, Ductility and Penetration Index for all the blends.

RAP/Virgin Ratio	Penetration	Softening Point (°C)	Ductility (cm)	Penetration Index	Viscosity at 135 °C Pa∙s	Viscosity at 160 °C Pa∙s
0/100	64	49.5	117	−0.743	0.40	0.10
30/70	45	54	89	−0.496	0.50	0.20
50/50	42	56	76	−0.385	0.60	0.24
100/0	22	62	17	−0.355	1.10	0.34

**Table 5:** Comparison of Marshall Properties of Control mix and RAP samples.

Properties	Control Mix	RAP 30	RAP 50	RAP 100	JKR Specifications
OBC (%)	5.00	5.00	5.00	5.00	-
BUW	2.424	2.373	2.392	2.405	-
Std	0.02	0.03	0.03	0.03	-
VIM (%)	3.39	4.5	4.3	4.14	3.0–5.0%
Std	0.12	0.3	0.25	0.26	-
VMA	15.16	16.93	17.03	15.82	-
Std	0.43	0.66	0.56	0.64	-
VFB (%)	77.63	68.08	67.55	73.83	70–80%
Std	1.10	1.15	1.39	1.10	-
Stability (kN)	12.69	14.83	17.48	22.89	>8 kN
Std	1.15	1.23	1.09	1.53	-
Flow, F (mm)	3.34	3.43	3.51	3.77	2.0–4.0 mm
Std	0.31	0.51	0.36	0.46	-
